# MicroRNA-331 and microRNA-151-3p as biomarkers in patients with ST-segment elevation myocardial infarction

**DOI:** 10.1038/s41598-020-62835-w

**Published:** 2020-04-03

**Authors:** Martin Horváth, Veronika Horváthová, Petr Hájek, Cyril Štěchovský, Jakub Honěk, Ladislav Šenolt, Josef Veselka

**Affiliations:** 1 Department of Cardiology, Charles University, 2nd Faculty of Medicine and Motol University Hospital, Prague, Czech Republic; 2 Faculty of Science, Charles University, Prague, Czech Republic; 3 Department of Rheumatology, Charles University, 1st Faculty of Medicine and Rheumatology Institute, Prague, Czech Republic

**Keywords:** miRNAs, Myocardial infarction, Atherosclerosis

## Abstract

We sought to analyse plasma levels of peripheral blood microRNAs (miRs) as biomarkers of ST-segment-elevation myocardial infarction (STEMI) due to type-1 myocardial infarction as a model situation of vulnerable plaque (VP) rupture. Samples of 20 patients with STEMI were compared both with a group of patients without angina pectoris in whom coronary angiogram did not reveal coronary atherosclerotic disease (no coronary atherosclerosis-NCA) and a group of patients with stable angina pectoris and at least one significant coronary artery stenosis (stable coronary artery disease-SCAD). This study design allowed us to identify miRs deregulated in the setting of acute coronary artery occlusion due to VP rupture. Based on an initial large scale miR assay screening, we selected a total of 12 miRs (three study miRs and nine controls) that were tested in the study. Two of the study miRs (miR-331 and miR-151-3p) significantly distinguished STEMI patients from the control groups, while ROC analysis confirmed their suitability as biomarkers. Importantly, this was observed in patients presenting early with STEMI, even before the markers of myocardial necrosis (cardiac troponin I, miR-208 and miR-499) were elevated, which suggests that the origin of miR-331 and miR-151-3p might be in the VP. In conclusion, the study provides two novel biomarkers observed in STEMI, which may be associated with plaque rupture.

## Introduction

Rupture of a vulnerable atherosclerotic plaque (VP), which leads to acute artery occlusion due to an overlying thrombosis, is a potentially devastating situation resulting in acute coronary syndromes (ACS), ischaemic stroke and other acute complications of atherosclerosis^1–5^. Early detection of VP *in vivo* is essential for effective primary prevention of their rupture, which might aid in the reduction of cardiovascular morbidity and mortality^6^. A potent biomarker that would be sensitive enough for the presence of a VP with a reasonable specificity could be a very important piece of this puzzle.

MicroRNAs (miRs) are small, non-coding RNA molecules that act as modifiers of gene expression^7–9^. Once they bind to their target mRNA, they may cause its degradation or suppression of its translation^7–9^. Thus, miRs control many cellular processes and play a role in the pathogenesis of various diseases that include atherosclerosis^7–9^. The molecules are very stable, easy to detect with quantitative polymerase chain reaction (qPCR) and are relatively tissue specific^9–12^. Due to these properties, miRs appear to be very suitable biomarkers.

The aim of this study was to identify plasma miRs from peripheral blood samples of patients with ST-segment-elevation myocardial infarction (STEMI) that might help quicken its diagnostics or may even be used directly as markers of VP. Such biomarkers might be used for the risk stratification of patients and both aid in tailoring the primary preventive measures and help as prognostic markers in patients with clinically manifested atherosclerosis.

## Results

### Baseline characteristics

A total of 60 patients were evenly divided between patients with STEMI (20 patients, 66.1 ± 9.5years, 85% men), patients with SCAD (20 patients, 65.2 ± 12.5 years, 65% men) and the NCA group (20 patients, 56.5 ± 12.9 years, 55% men). Baseline characteristics of the study population are summarized in Table 1.

**Table 1:** Here we provide the baseline characteristics of all three study groups.

	STEMI	SCAD	NCA	STEMI vs. SCAD (p-values)	STEMI vs. NCA (p-values)	SCAD vs. NCA (p-values)
Sex (male)-N(%)	17 (85)	13 (65)	8 (40)	0.273	0.008*	0.122
Age (mean ± SD)	66.1 (±9.5)	65.2 (±5)	57.9 (±12.9)	0.789	0.027*	0.077
BMI (mean ± SD)	29.6 (±6.8)	30.1 (±4.7)	29.3 (±4.6)	0.779	0.897	0.606
Arterial hypertension-N (%)	12 (60)	17 (85)	12 (60)	0.155	1.000	0.155
Dyslipidaemia-N (%)	6 (30)	15 (75)	11 (55)	0.010*	0.200	0.320
Diabetes mellitus-N (%)	8 (40)	9 (45)	3 (15)	1.000	0.155	0.082
Smoking-N (%)	13 (65)	11 (55)	4 (20)	0.748	0.010*	0.048*
Stroke-N (%)	1 (5)	2 (10)	1 (5)	1.000	1.000	1.000
Statin-N (%)	2 (10)	14 (70)	11 (55)	0.0002*	0.006*	0.515
ASA-N (%)	11 (55)	17 (85)	12 (60)	0.082	1.000	0.155
Clopidogrel-N (%)	0 (0)	10 (50)	1 (5)	0.0001*	1.000	0.003*

The data that significantly differ between the groups are indicated with an asterisk. *Twenty patients were enrolled in each group. Normally distributed data are presented as means ± standard deviation (±SD) and non-normally distributed data as medians with interquartile range (IQR). The distribution of the data was evaluated using the D’Agostino and Pearson omnibus normality test, the Shapiro-Wilk normality test and the Kolmogorov-Smirnov normality test. The differences in the background clinical data between the study groups were evaluated using the Student’s t-test.

### Safety and feasibility

No complications of the sample collection and evaluation were noted. All STEMI patients as well as all patients with SCAD were treated with a percutaneous coronary intervention (PCI). No complications of the diagnostic angiography or PCI were observed.

### Markers of acute coronary syndrome

**Figure 1:**
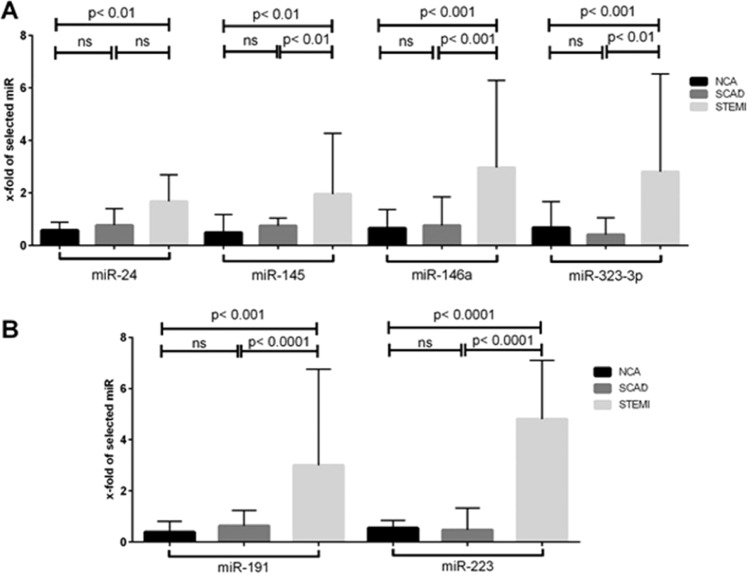
Amongst the miRs that were se1elected as positive controls, four (miR-146a, miR-145, miR-24 and miR-323p) were significantly up-regulated in STEMI. (**A**) The relative expressions of miRs associated with platelet activation (miR-223 and miR-191) were significantly higher in patients with STEMI. (**B**) There were twenty patients enrolled in each of the three study groups. For the purposes of the comparison between the relative expressions of miRs in the three study groups, the Kruskal-Wallis one-way analysis of variance was used.

Amongst the miRNAs associated with ACS, which were se1elected as positive controls, four (miR-146a, miR-145, miR-24 and miR-323p) were significantly up-regulated in STEMI: miRNA-146a [STEMI: 2.970 (1.405–6.280) vs. SCAD: 0.760 (0.153–1.845), p < 0.001, STEMI: 2.970 (1.405–6.280) vs. NCA: 0.660 (0.175–1.370), p < 0.001]; miRNA-145 [STEMI: 1.955 (1.025–4.270) vs. SCAD: 0.750 (0.223–1.038), p < 0.01, STEMI: 1.955 (1.025–4.270) vs. NCA: 0.490 (0.193–1.178), p < 0.01]; miRNA-24 [STEMI: 1.675 (0.750–2.693) vs. SCAD: 0.765 (0.185–1.405), p = ns; STEMI: 1.675 (0.750–2.693) vs. NCA: 0.575 (0.105–0.883), p < 0.01] and miRNA-323p [STEMI: 2.805 (1.268–6.533) vs. SCAD: 0.405 (0.353–1.058), p < 0.01; STEMI: 2.805 (1.268–6.533) vs. NCA: 0.675 (0.300–1.670), p < 0.001] (Fig. 1A). MicroRNA-155 was not expressed in any of the study groups.

### Markers of myocardial necrosis

Both of the miRs that were tested as markers of myocardial necrosis (miR-208 and miR-499) were not expressed in any of the study groups. The median level of high-sensitivity troponin I (hsTnI) did not exceed the cut-off value for myocardial infarction in the STEMI patients at the time of blood sample collection (median hsTnI in STEMI 107.6 ng/l). The median time from the onset of chest pain to blood sample collection in STEMI was 2.25 hours.

### Markers of platelet activation

The relative expressions of miRs associated with platelet activation were significantly higher in patients with STEMI: miR-223 [STEMI: 4.810 (1.560–7.100) vs. SCAD: 0.475 (0.165–1.328); p < 0.0001; STEMI: 4.810 (1.560–7.100) vs. NCA: 0.550 (0.270–0.850); p < 0.0001] and miR-191 [STEMI: 3.000 (1.473–6.758) vs. SCAD: 0.630 (0.218–1.238); p < 0.001; STEMI: 3.000 (1.473–6.758) vs. NCA: 0.390 (0.163–0.808); p < 0.0001] (Fig. 1B).

### Results of the study microRNAs

Amongst the study miRs, miR-331 and miR-151-3p, were significantly up-regulated in patients with STEMI. MicroRNA-518d was not deregulated in any of the study groups.

**Figure 2:**
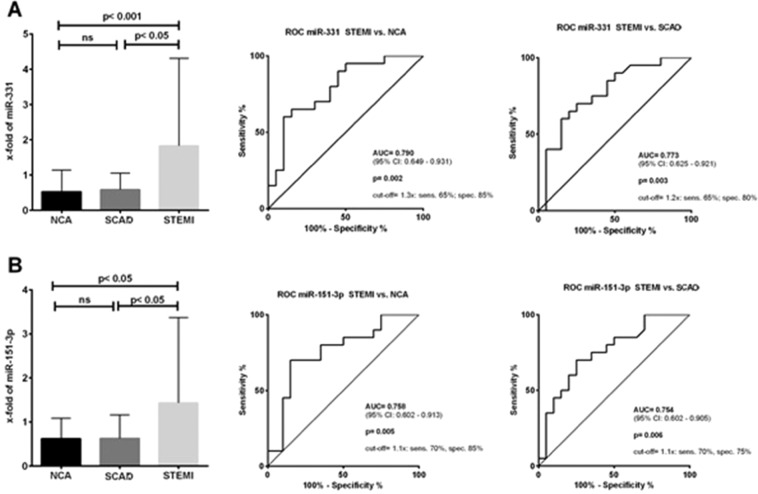
MicroRNA-331 distinguished patients with STEMI from both control groups. (**A**) The ROC analysis confirmed the suitability of miR-331 as a biomarker. (**A**) Plasma levels of miR-151-3p were also significantly higher in patients with STEMI when compared to the control groups. (**B**) The ROC analysis yielded a promising sensitivity and specificity for the differentiation of STEMI from both control groups (**B**).

MicroRNA-331 distinguished patients with STEMI from both control groups [STEMI: 1.830 (0.775–4.313) vs. SCAD: 0.585 (0.243–1.050); p < 0.05; STEMI: 1.830 (0.775–4.313) vs. NCA: 0.525 (0.176–1.140); p < 0.01] (Fig. 2A). The ROC analysis confirmed the suitability of miR-331 as a biomarker (STEMI vs. NCA: AUC = 0.790 [95% CI; 0.649–0.931], p = 0.002; STEMI vs. SCAD: AUC = 0.773 [95% CI; 0.625–0.921], p = 0.003) (Fig. 2A). The results suggest a sensitivity of 65% and specificity of 85% for distinguishing STEMI patients from NCA with a cut-off value of 1.3x. Alternatively, the sensitivity was 65% and the specificity was 80% for separating STEMI from SCAD patients with a cut-off value of 1.2x (Fig. 2A).

The plasma levels of miR-151–3p were also significantly higher in patients with STEMI when compared to the control groups [STEMI: 1.430 (0.770–3.370) vs. SCAD: 0.625 (0.223–1.163); p < 0.05; STEMI: 1.430 (0.770–3.370) vs. NCA: 0.620 (0.243–1.083); p < 0.05] (Fig. 2B). The ROC analysis confirmed a good power to distinguish STEMI from the control groups (STEMI vs. NCA: AUC = 0.758 [95% CI; 0.602–0.931], p = 0.005; STEMI vs. SCAD: AUC = 0.754 [95% CI; 0.602–0.905], p = 0.006) (Fig. 2B). The sensitivity and specificity for the detection of STEMI patients when compared with NCA was 70% and 85% respectively with a cut-off value 1.1x. When using miR-151-3p to differentiate STEMI from SCAD the sensitivity was 70%, the specificity was 75% usingthe cut-off value was 1.1x (Fig. 2B).

An analysis of miR-151-3p and miR-331 was also perfomed. The combination of the two miR did not provide a better power to predict STEMI from the control groups in a ROC analysis (STEMI vs. NCA: AUC = 0.790; STEMI vs. SCAD: AUC = 0.627).

## Discussion

The principle findings of the study may be summarized as follows: (1) most important finding of this research is the newly described association between the plasma levels of miR-331 and miR-151-3p and STEMI, (2) the results of the positive control miRs demonstrate that the methodology in this pilot study was executed properly, 3) the results suggest that the source of miR-331 and miR-151-3p is outside of the myocardium since the markers of myocardial necrosis were still negative at the time of sampling and 4) the platelet-derived miRs were elevated in STEMI, which indicates that the STEMI patients suffered from a type-1 myocardial infarction (T1 MI) due to a rupture of a VP^13^.

Little is known about the molecular biology of miR-331. Its deregulation has been linked to the pathogenesis of several types of human cancer^14–17^. Its down-regulation was observed in a very small study of human abdominal aortic aneurysm specimens^18^. Several observational studies have proposed its expression in macrophages^19,20^. Interestingly, this miR is up-regulated in many types of leukaemia, including acute myeloid leukemia, indicating its possible association with the monocyte-macrophage system^14,17^. A study conducted on patients with chronic lymphocytic leukaemia found an up-regulation of miR-331^14^. The authors described a possible association between this miR and the suppressor of cytokine signalling 1 (SOCS1) protein, an inhibitor of the Janus kinase/signal transducer and activator of transcription (JAK/STAT) pathway^14^. This may be one of the possible links between the observed over-expression of miR-331 and STEMI. Up-regulation of SOCS1 has been identified as an anti-inflammatory mechanism in atherosclerosis^21^. MicroRNAs have the ability to block SOCS1, leading to a pro-inflammatory response in atherosclerotic plaques^22^. This pathophysiological mechanism has been previously described with miR-155, which is however the only positive control that was not deregulated in the present study^22^. Another possible link between miR-331 and VP rupture might be its proposed impact on the phosphatidylinositol 3-kinase/protein kinase B (PI3K/AKT) signalling pathway, which has a role in the stabilization of VP^15,23,24^.

The evidence about miR-151-3p is even scarcer. Several studies have observed a relationship with cancer^25,26^. Its association with atherosclerosis has not been reported to date. Liu *et al*. observed an interaction between miR-151-3p and STAT3, which regulates the inflammatory response in macrophages^27^. This is a plausible explanation of its deregulation in STEMI. Moreover, the dependence of miR-151-3p expression on the amount of endothelial shear stress, a well-known modifier of atherosclerotic plaque progression and destabilization, has also been described^28–31^. Another possible explanation of its deregulation might be an association with type 2 diabetes mellitus, although its prevalence was similarly high in the STEMI and SCAD groups^32^.

This study established the association between four previously described miRs (miR-146a, miR-145, miR-24 and miR-323p) and ACS confirming the well-executed methodology. The data are quite unique because they were obtained in a study with very well-defined groups. A more general definition of ACS was often used in the previously published research in order to simplify patient recruitment^12,33^. Patients with non-ST segment elevations myocardial infarction or unstable angina pectoris were frequently included, which may be a source of inaccuracies when we focus directly on markers of VP rupture^12,33^. Furthermore, all patients enrolled in this study, including the negative controls, had a well-defined coronary anatomy by coronary angiography and well-defined background clinical data. Another fact that differentiates our data from previous research is that the levels of miRs were determined from peripheral venous blood samples rather than samples obtained directly from the coronary arteries or the coronary sinus^12,33^. This may certainly lead to an inability to detect small differences in miR levels. However, we believe that this approach may be much better convertible into common practice.The sole positive control that was not upregulated in the study was miR-155.A possible explanation for this inconsistency might be that the data about this miR are somewhat uncertain and contradictory^7,34^. While some studies suggest that its upregulation may promote atherosclerosis, others suggested that it may have a protective role or no effect at all. The decision to use this miR as a positive control was thus to some extent unfortunate^34^.

The levels of myocardial enriched miR-208 and miR-499 were not significantly elevated in STEMI patients at the time of blood sample collection^35^. The median level of hsTnI in STEMI was below the cut-off value for myocardial infarction at the time of sample collection. These facts suggest that the patients enrolled in the STEMI group presented to the hospital before myocardial injury would compromise the results of a study designed to detect miRs associated with VP rupture. All STEMI patients had an acute arterial occlusion due to T1 MI confirmed on coronary angiography. Since rupture of a thin-cap fibroatheroma is the most common cause of VP associated coronary artery thrombosis, we hypothesize that the study miRs may directly be associated with the presence of such atherosclerotic lesions^36,37^. Clearly, this hypothesis remains to be tested in larger studies using invasive imaging techniques.

The plasma levels of the platelet derived miR-223 and miR-191 were elevated in STEMI. This suggests that the reason for the acute arterial occlusionwas the formation of an intracoronary thrombus in the setting of a T1 MI. It also suggests an alternative explanation for the elevation of the study miR-331 and miR 151-3p, since their origin might be in activated platelets. However, no association between both of the study miRs and platelets has been noted to date^38,39^. Importantly, the study miRs could serve as biomarkers of ACS even if their origin was in platelets. Platelet-derived miRs can directly affect gene expression in their neighbouring cells including the endothelium^40^. Such miRs have indeed been proposed as potential prognostic markers in atherosclerosis^41^.

Present study has several limitations and should thus be appreciated as a pilot project aiming to generate hypotheses for further research. The most obvious limitations include its observational design and the relatively small study sample^12,33^. This did not allow us to reliably verify whether the level of study miRs could be influenced by some possible confounding factors that were not evenly balanced between the patient groups. The differences in baseline clinical data are provided in Table 1. These are mainly due to the different distribution of characteristics in NCA, which is based on the very indication of coronary angiography in these patients. The relationship between the levels of peripheral blood miRs and age has been well-described before, and sex differences should not significantly influence the results^42^. There was no correlation between the level of miR-151-3p of miR-331 with age in any of the groups. We also did not find any statistically significant difference between the levels of both miR-151-3p and miR-331 between sexes in all of the groups. The combination of the two markers as a signature did not yield better results in our study. The reason for this might be the limited study sample and the close correlation between the markers. The prevalence of diabetes mellitus was lower among NCA, which could affect the levels of both study miRs, especially miR-151-3p where an association has previously been described^32^. If we assume that the origin of the study miRs might be in platelets, their levels could also be influenced by anti-platelet therapy, which was also unevenly distributed between the study groups^38,39^. We believe that these limitations did not corrupt the results. The hypotheses provided in the study will be tested in larger studies which will account for the potential confounders and will also provide more insight with invasive imaging techniques.

In conclusion, the study provides two novel biomarkers observed in STEMI, which may be associated with plaque rupture.

## Methods

### Study design and population

A total of 60 patients who underwent coronary angiography (CAG) at the same institution were enrolled in this case-control observational study. The study population was divided evenly between a cohort of 20 patients with STEMI and two control groups. These were introduced in the study in order to determine whether the studied miRs are not only markers of atherosclerosis in general (STEMI vs. NCA), but rather potential markers of patients with VPs (STEMI vs. SCAD).

All patients included in the study were ≥18 years old and provided signed informed consent. Patients in the STEMI cohort met the definition according to the European Society of Cardiology guidelines (i.e. patients with persistent chest discomfort or other symptoms suggestive of ischaemia and significant ST-segment elevation in at least two contiguous leads) and had a proven coronary artery occlusion as a culprit on CAG^43^. The NCA cohort consisted of patients without angina pectoris who underwent a clinically indicated coronary angiography that did not reveal any atherosclerotic affection of the coronary arteries. In most cases, these were patients with valvular heart disease. The SCAD consisted of patients with stable angina pectoris and at least one significant coronary artery stenosis (more than 50% stenosis) proven by CAG. The study was approved by the Ethics committee of the Motol University Hospital under the reference number EK-1158/18. All research was performed in accordance with the relevant guidelines and regulations.

### Plasma collection and storage

Peripheral blood samples were collected into 9 ml EDTA tubes. In patients with STEMI, the blood was collected immediately after the admission of the patient to the hospital. Blood samples of SCAD patients were collected after a significant stenosis was revealed, but always before a PCI was performed. The blood samples of NCA patients were collected after the diagnostic CAG yielded a negative result.

Immediately after the collection, the whole blood samples were centrifuged at 1000 *g* for 10 minutes to separate plasma from red blood cells. Next, the plasma samples were transferred into DNase/RNase free tubes and centrifuged once again at 2000 *g* for 15 minutes in order to remove platelets from the sample. Finally, the resultant plasma was aliquoted per 500 μl into DNase/RNase free Eppendorf tubes and stored at −80 °C.

### Total RNA isolation and its quantification

Total RNA was isolated from 100 μl plasma samples using the miRNeasy Serum/Plasma kit (Qiagen, Hilden, Germany) according to the manufacturer’s protocol and stored at −80 °C.

### Initial TaqMan screening

In order to identify previously unknown miRs that could be associated with VP, we performed an initial screening of a large number of miRs. From the isolated total RNA, we screened miRs which were differently expressed between a group of four patients with STEMI and a group of four NCA using TaqMan Array microRNA Cards (Thermo Fisher Scientific, Waltham, MA, USA). Screening of miRs in plasma samples started with reverse transcription (RT) using Megaplex™ RT Primers, Human Pool A + B v2.1 (Thermo Fisher Scientific, Waltham, MA, USA) with TaqMan MicroRNA Reverse Transcription Kit (Thermo Fisher Scientific, Waltham, MA USA), followed by pre-amplification reaction using MegaplexPreAmp Primers (Thermo Fisher Scientific, Waltham, MA, USA) and TaqMan® PreAmp Master Mix (Thermo Fisher Scientific, Waltham, MA, USA), finished with quantitative polymerase chain reaction (qPCR) using TaqMan Array Human MicroRNA A + B Cards (Thermo Fisher Scientific, Waltham, MA, USA) with TaqMan Universal PCR Master Mix, no AmpErase UNG (Thermo Fisher Scientific, Waltham, MA, USA) according to the company-provided protocol. The mean of all expressed miRs in TaqMan Array Human MicroRNA A + B Cards was used for the normalization of screened miRs.

Based on the results of the screening, we selected a total of 12 miRs for validation on the whole study population. Firstly, we selected three study miRs that were significantly deregulated in the initial screening (miR-331, miR-151-3p and miR-518d) and have not yet been associated with cardiovascular disease. We further selected miR-146a, miR-145, miR-155, miR-24 and miR-323p, which were previously linked to ACS, as positive controls^44–49^. MicroRNA-208 and miR-499 associated with myocardial necrosis were selected as controls of timely sampling, since our aim was to detect miRs associated with plaque rupture rather than myocardial necrosis^35^. Lastly, we selected miR-191 and miR-223 that are associated with platelet activation to prove the formation of coronary thrombus due to T1 MI in STEMI^41^.

### Study miR analysis

The selected miRs were first reverse transcribed by particular TaqMan microRNA Assays (Thermo Fisher Scientific, Waltham, MA, USA) with TaqMan MicroRNA Reverse Transcription Kit (Thermo Fisher Scientific, Waltham, MA, USA) followed by qPCR reaction using specific TaqMan microRNA Assays (Thermo Fisher Scientific, Waltham, MA, USA) with TaqMan Universal PCR Master Mix, no AmpErase UNG (Thermo Fisher Scientific, Waltham, MA, USA). For all reverse transcription (RT) reactions, thermocycler Lab Cycler (Sensoquest, Göttingen, Germany) was used and all quantification reactions were perfomed using the QuantStudio 7 Flex Real-Time PCR System (Thermo Fisher Scientific, Waltham, MA, USA). Spike-in control cel-miR-39 (IDT, San Jose, CA, USA), originating from *Caenorhabditis elegans*, was used for the normalization of cell-free miRs. For calculation of miRNA levels was used 2^−ΔΔCt^ method determining fold change in miRNA expressions of the patient group relative to the control groups^50^.

### Heparinase I treatment

Patients with STEMI were treated with heparin prior to the admission to the hospital and the collection of blood samples. Since, heparin is a well-known inhibitor of RT and qPCR reactions, we incubated all STEMI samples with 0.3 U of heparinase I from *Flavobacterium heparinum* (Sigma-Aldrich, St. Louis, MO, USA) per 10 ng of total RNA at 26 °C for 1 h before the RT reaction, according to the protocol published by Li and colleagues^51–54^.

### Statistical analysis

Normally distributed data are presented as means ± standard deviation (±SD) and non-normally distributed data as medians with interquartile range (IQR). The distribution of the data was evaluated using the D’Agostino and Pearson omnibus normality test, the Shapiro-Wilk normality test and the Kolmogorov-Smirnov normality test. The differences in the background clinical data between the study groups were evaluated using Student’s t-test. For the purposes of the comparison between the relative expressions of miRs between the three study groups, the Kruskal-Wallis one-way analysis of variance was used. The power of the study miRs to predict STEMI was analysed by the receiver operating characteristic (ROC) curve analysis; the area under the curve (AUC) was calculated with 95% confidence intervals (CI). A p-value of ≤0.05 was considered to indicate a statistically significant difference. The statistical analyses were performed using GraphPad Prism version 6 (La Jolla, CA, USA). We used Binary logistic regression model to combine the levels of miR-151-3p and miR-331 as a signature. The combined probability model was then used to assess the power of this signature to predict patients with STEMI from the control groups in an ROC analysis. The IBM SPSS Statistics for Windows, Version 25.0 (IBM Corp., Armonk, NY) was used solely for this analysis.
